# Acute migraine therapy with external trigeminal neurostimulation (ACME): A randomized controlled trial

**DOI:** 10.1177/0333102418811573

**Published:** 2018-11-17

**Authors:** Denise E Chou, Marianna Shnayderman Yugrakh, Dana Winegarner, Vernon Rowe, Deena Kuruvilla, Jean Schoenen

**Affiliations:** 1 Department of Neurology, Columbia University Medical Center, New York, NY, USA; 2 Rowe Neurology Institute, Lenexa, KS, USA; 3 Department of Neurology, Yale University School of Medicine, New Haven, CT, USA; 4 Headache Research Unit, University Department of Neurology CHR, Citadelle Hospital, Liege, Belgium

**Keywords:** Migraine, external trigeminal nerve stimulation, acute treatment, neuromodulation

## Abstract

**Objective:**

To assess the safety and efficacy of external trigeminal nerve stimulation for acute pain relief during migraine attacks with or without aura via a sham-controlled trial.

**Methods:**

This was a double-blind, randomized, sham-controlled study conducted across three headache centers in the United States. Adult patients who were experiencing an acute migraine attack with or without aura were recruited on site and randomly assigned 1:1 to receive either verum or sham external trigeminal nerve stimulation treatment (CEFALY Technology) for 1 hour. Pain intensity was scored using a visual analogue scale (0 = no pain to 10 = maximum pain). The primary outcome measure was the mean change in pain intensity at 1 hour compared to baseline.

**Results:**

A total of 109 participants were screened between February 1, 2016 and March 31, 2017. Of these, 106 patients were randomized and included in the intention-to-treat analysis (verum: *n* = 52; sham: *n* = 54). The primary outcome measure was significantly more reduced in the verum group than in the sham group: −3.46 ± 2.32 versus −1.78 ± 1.89 (*p* < 0.0001), or −59% versus −30% (*p* < 0.0001). With regards to migraine subgroups, there was a significant difference in pain reduction between verum and sham for ‘migraine without aura’ attacks: mean visual analogue scale reduction at 1 hour was −3.3 ± 2.4 for the verum group versus −1.7 ± 1.9 for the sham group (*p* = 0.0006). For ‘migraine with aura’ attacks, pain reduction was numerically greater for verum versus sham, but did not reach significance: mean visual analogue scale reduction at 1 hour was −4.3 ± 1.8 for the verum group versus −2.6 ± 1.9 for the sham group (*p* = 0.060). No serious adverse events were reported and five minor adverse events occurred in the verum group.

**Conclusion:**

One-hour treatment with external trigeminal nerve stimulation resulted in significant headache pain relief compared to sham stimulation and was well tolerated, suggesting it may be a safe and effective acute treatment for migraine attacks.

**Study protocol:**

ClinicalTrials.gov Identifier: NCT02590939.

## Introduction

Current available acute migraine treatments are mainly pharmacologic therapies (e.g. analgesics, non-steroidal anti-inflammatory drugs (NSAIDs), and ‘migraine-specific’ drugs such as ergots and triptans) (1–3) that have incomplete efficacy, as well as several side effects and contraindications; their excessive intake may also lead to medication overuse headache and chronification of migraine (4,5). These limitations highlight the need for non-pharmacological options for acute migraine treatment.

External trigeminal nerve stimulation (e-TNS) was initially found to produce a sedative effect (6) and subsequently demonstrated safety and efficacy in the prevention of episodic migraine (7). Tolerability and patient satisfaction with e-TNS for migraine prevention have been confirmed by a prospective study on 2313 patients (8) and in several recent studies (9–15). Regarding the use of e-TNS in the acute treatment of migraine, a few small pilot studies (16,17) and a post-marketing survey in Europe (18) suggested a possible therapeutic effect. Recently, an open-labeled pilot trial showed an average reduction of headache pain severity by 57.1% after a 1-hour e-TNS treatment, with 76.7% of patients reporting ≥50% pain relief (19). Based on these findings, we sought to further evaluate the safety and efficacy of e-TNS for acute pain relief during migraine attacks via the ACME study (ACute treatment of Migraine with External trigeminal nerve stimulation): a randomized, double-blind, sham-controlled trial.

## Methods

### Standard protocol approvals and patient consents

The study protocol was reviewed and approved by the Institutional Review Boards (IRBs) at Columbia University Medical Center (serving as the central IRB for Columbia and Rowe Neurology Institute) and Yale University School of Medicine. Written informed consent was obtained from all participants.

### Study design

The ACME study was a prospective, double-blind, randomized, sham-controlled clinical trial conducted across three headache centers in the United States (Columbia University Medical Center – NY, Yale University School of Medicine – CT, and Rowe Neurology Institute – KS).

### Patients

Male or female adults (aged 18–65 years) were eligible for enrollment if they had a diagnosis of migraine with or without aura (according to International Headache Society criteria ICHD-III beta (2013) section 1) and were experiencing a migraine attack with or without aura, with headache lasting for at least 3 hours and pain severity stable for at least 1 hour prior to enrollment; subjects may have used any acute medications to treat the attack, but not within the 3 hours before enrollment. The requirement for a minimum headache duration of 3 hours prior to enrollment was implemented to enable, as much as possible, a stable pain intensity at baseline and to minimize the potential for spontaneous headache remission. Exclusion criteria were the following: pregnancy; treatment with botulinum toxin to the head in the prior 4 months; supraorbital nerve blocks in the prior 4 months; diagnosis of other primary or secondary headache disorders, except of medication overuse headache; headache location not involving the frontal, retro- or peri-orbital regions; forehead skin allodynia; use of opioid medications; intake of acute migraine medication within the 3 hours prior to enrollment; implanted metal or electrical devices in the head; cardiac pacemaker or implanted or wearable defibrillator; or previous experience with e-TNS.

### Procedures

**Figure 1.**
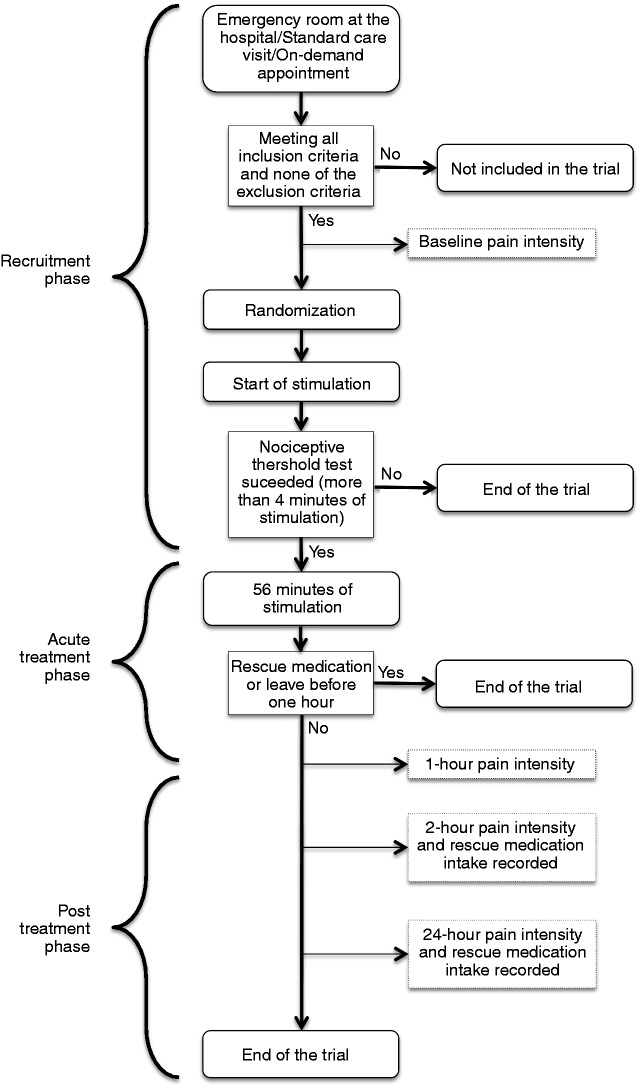
Study flowchart.

Patients were recruited at the three centers through the following means: (i) during a routine outpatient office visit; (ii) at the clinic’s or hospital’s urgent care headache unit; or (iii) during an on-demand appointment arranged at the time of a qualifying migraine attack (for patients having been informed about the study beforehand at a standard care visit or via advertisement). Recruited patients were randomly assigned to receive a 1-hour e-TNS session with verum or sham stimulation at the study site. Headache pain intensity was measured using a visual analogue scale (VAS) following the International Headache Society guidelines for controlled trials of drugs in migraine (20). Patients reported their pain intensity score using an 11-point (0 = no pain to 10 = maximum pain) VAS before the treatment (baseline score during the recruitment phase), immediately after the 1-hour treatment session (acute treatment phase), and at 2 hours and 24 hours after the beginning of treatment initiation (post-treatment phase). Use of migraine rescue medications was not permitted during the 2-hour time period from the beginning of the treatment (i.e. the 1-hour e-TNS session followed by 1 hour of observation); following this interval, patients were permitted to use rescue medication and their intake was recorded for 24 hours from the start of the e-TNS treatment. Adverse events (AEs) were monitored by the investigators throughout the 24-hour period. The overall study flow is illustrated in Figure 1. Subjects who could not bear the sham or verum paresthesia sensation during the first 4 minutes of the e-TNS session (‘nociceptive threshold test’) were considered as having a low nociceptive threshold likely due to forehead skin allodynia; in such cases, e-TNS was discontinued if the patients desired for their comfort.

### Neurostimulation

**Figure 2.**
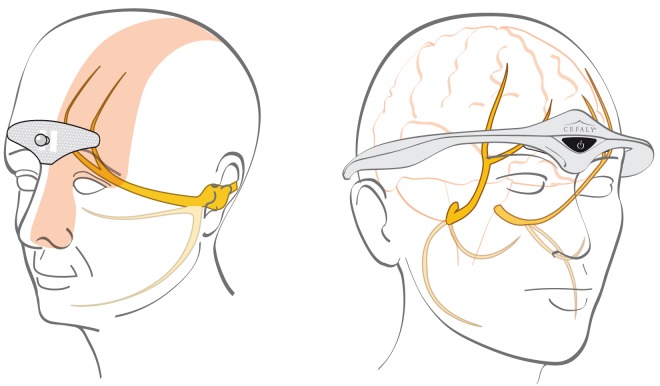
Electrode positioning: (*left*) the electrode covers the supratrochlearis and supraorbitalis nerves and (*right*) the neurostimulator device is placed on the forehead and connected to the electrode.

Neurostimulation was applied via the e-TNS Cefaly® device (CEFALY Technology, Seraing, Belgium) for a 1-hour session. The device is a constant current generator for a maximum skin impedance of 2.2 kΩ that delivers rectangular biphasic symmetrical pulses with a zero electrical mean. The pulse frequency used in the current study for the verum device is 100 Hz and pulse width is 250 µs; the total maximum dose of current delivered by a 1-hour session is 1.284 C. The intensity increases linearly to reach a maximum of 16 mA after 14 minutes and then remains constant for 46 minutes. The electrical pulses are transmitted transcutaneously via a supraorbital bipolar self-adhesive electrode (30 × 94 mm) placed on the forehead, designed to cover and excite (trigger action potentials) the supratrochlearis and supraorbitalis nerves bilaterally (Figure 2).

### Randomization and masking

Patients were randomly assigned to a verum or sham e-TNS device. The devices were sequentially numbered following a random allocation sequence generated by the R&D department of the device manufacturer and stratified by center with a 1:1 allocation to one of the two treatment groups (verum or sham) using a block size of 4. Details of the allocated group were sent in a sealed envelope to the coordinating investigator. All site investigators and research staff, as well as patients, remained blinded to the device identities throughout the course of the study. The sham device was strictly identical in shape and color to the verum device, along with identical beeping and flashing. It was not possible for the patient nor for the investigator (who enrolled patients, assigned the device, and collected outcome measurements) to distinguish which device was verum or sham. Both devices used identical rectangular biphasic symmetrical pulses of 250 µs width that induced paresthesia: the sham device via low-frequency pulses of 3 Hz and the verum device via high-frequency pulses of 100 Hz (6).

### Outcome measures

The primary outcome measure was the mean change in pain score at the 1-hour time point compared to baseline. Secondary outcome measures were the following: mean change in pain score at the 2-hour and 24-hour time points compared to baseline, as well as the proportion of subjects not having used migraine rescue medication at 2 hours and within 24 hours after initiation of the e-TNS session. The primary and secondary outcome measures were selected to determine if e-TNS would be effective in providing headache pain relief, though not necessarily to completely abort a migraine attack.

Exploratory outcome measures were the following: proportion of subjects pain-free at 1-hour, 2-hour, and 24-hour time points; proportion of subjects with ≥30% pain relief at 1-hour, 2-hour, and 24-hour time points; proportion of subjects with ≥50% pain relief at 1-hour, 2-hour, and 24-hour time points. A safety assessment was based on the number of reported AEs and their severity. No outcome measures were assessed for sedation.

### Statistical analysis

Results of our recent, open-labeled pilot trial on the acute treatment of migraine using e-TNS showed a reduction in mean pain score of 59% after 1 hour and 55% after 2 hours (19); the standard deviation related to the mean pain score (using the 11-point VAS) was 2.42 after 1 hour and also after 2 hours. In a previous controlled trial with an acute treatment of migraine (diclofenac potassium) (21), there was a reduction in mean pain score of 10% after 1 hour and 14% after 2 hours in the placebo group. Based on the above, we assumed a difference in mean pain score (using the 11-point VAS) of 2.83 after 1 hour and 2.40 after 2 hours between the verum and sham groups. Consequently, a minimum of 17 patients in each group was statistically required to detect a significant difference in mean pain score after 1 hour and after 2 hours, with a power of 80% and a two-sided alpha level of 5% using a *t*-test for two independent samples.

Another placebo-controlled acute migraine study had previously reported use of rescue medication in 71% of subjects from the placebo group, compared to 41.33% for the active treatment group on average (22). Considering these percentages, 43 subjects in each group for our current study would be statistically required to detect a significant difference in rescue medication use after 24 hours, with a power of 80% and a two-sided alpha level of 5%, using a proportion test for two independent samples. Given that at least 43 subjects in each group were needed overall to detect a significant difference between the verum and sham groups in the 1-hour and 2-hour mean pain scores, as well as in rescue medication use, we determined the minimum number in each group to be at least 45 and therefore a total minimum recruitment of 90 subjects.

All relevant general, safety and efficacy data were descriptively summarized at each time point of the study. Continuous data were summarized by the number of patients (*n*), the arithmetic mean and the standard deviation. Categorical data were summarized by absolute (*n*) and relative (%) frequency tables. Within the same group (verum or sham), comparison between baseline and treatment results was performed using the Wilcoxon signed-rank test for paired samples. Comparison between the verum and sham groups was performed using the Mann–Whitney test for the outcomes related to change in pain score and the Fisher’s exact test for the outcomes related to proportion of patients. Statistical analysis was carried out on an intention-to-treat (ITT) basis (i.e. including all patients who gave their consent to participate to the study and who were randomized) using R statistical software (version 3.3.2; R Foundation for Statistical Computing, Vienna, Austria). Two-tailed *p*-values were computed with a cut-off for statistical significance set to 0.05. If a subject used rescue medication, pain scores following the medication intake were not included in the analysis; in such cases, the last value carried forward method was used for data imputation. In order to account for possible imbalance in baseline characteristics between the verum and sham groups, a post hoc statistical analysis was performed for the difference in mean VAS scores at 1 hour (primary outcome) in both groups using an analysis of covariance (ANCOVA) model with covariates of baseline VAS score, treatment, site, sex, migraine type (with or without aura), migraine duration, and prior acute medication use.

## Results

**Figure 3.**
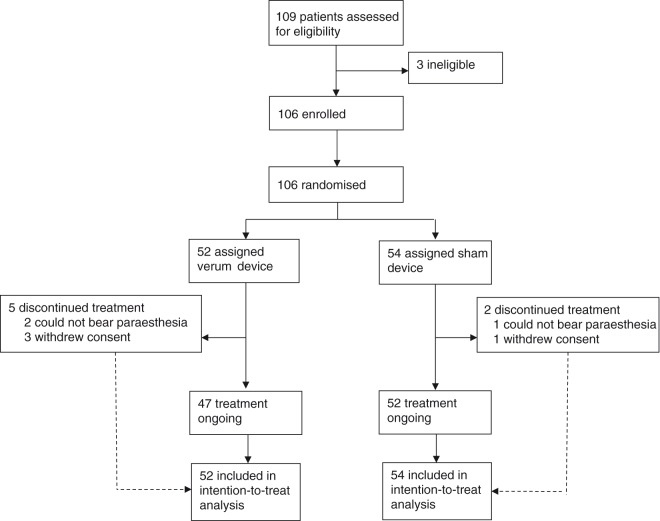
Trial profile.

Between February 1, 2016 and March 31, 2017, 109 patients were screened, of whom 106 met eligibility criteria, were randomly assigned to a treatment group (52 in the verum group and 54 in the sham group), and therefore eligible for the ITT analysis. Of these 106 randomized patients, seven subjects did not receive the full 1-hour e-TNS treatment: three patients (two verum and one sham) could not bear the paresthesia feeling and requested the stimulation be discontinued before the first 5 minutes elapsed, and four patients (three verum and one sham) withdrew from the study and stopped the stimulation before the end of the 1-hour e-TNS session. Therefore, a total of 99 randomized patients (47 verum and 52 sham) completed the 1-hour treatment phase and provided their pain scores at baseline and at the 1-hour time point. The trial profile of all patients is presented in Figure 3, and the repartition of patients within the two groups (verum or sham), as well as the corresponding demographic baseline characteristics, are presented in Table 1.

**Table 1.** Repartition of patients within the two groups (verum and sham) and corresponding demographic baseline characteristics.

	Verum	Sham	All
*n*	52	54	106
Age, years	39.71 ± 13.62	40.09 ± 12.65	39.90 ± 13.14
Sex			
Male	9 (17)	5 (9)	14 (13)
Female	43 (83)	49 (91)	92 (87)
Migraine with aura	12 (23)	5 (9)	17 (16)
Migraine without aura	40 (77)	49 (91)	89 (84)
Migraine attack duration before treatment, in hours, median [range]	7.00 [4.00–48.00]	6.00 [4.63–20.75]	6.00 [4.00–24.00]
Number of subjects who treated the current migraine attack with an acute medication prior to enrollment	17 (33)	14 (26)	31 (29)

Data are expressed as *n* (%), mean ± standard deviation, or median [interquartile range].

**Figure 4.**
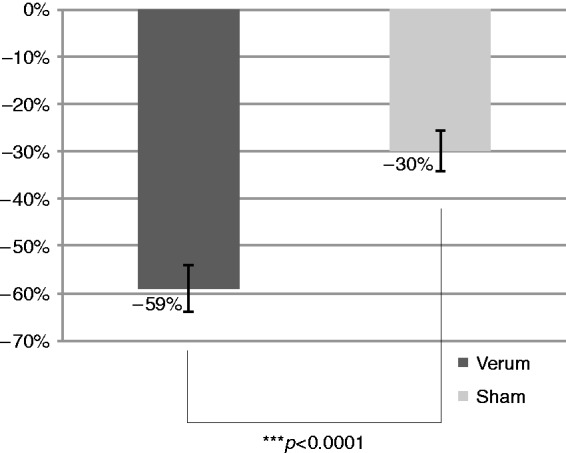
Relative change in pain intensity at 1 hour.

Outcome measures are detailed in Table 2. The primary outcome (mean change in pain score at 1 hour compared to baseline) was significantly decreased (*p* < 0.0001) in the verum and sham groups, but much more in the verum (−59%) than in the sham group (−30%); the effect size was large, with a Cohen’s *d* value of 0.88 (Figure 4). Applying the aforementioned post hoc ANCOVA sensitivity analysis, the treatment effect defined by the primary outcome measure remained highly significant (*p* < 0.0001).

**Table 2.** Study outcomes.

	Verum	Sham	Comparison between groups
*n*	52	54	
**VAS scores**			
VAS baseline	5.92 ± 1.68	6.17 ± 1.81	
VAS 1 hour	2.46 ± 2.23	4.39 ± 2.44	
VAS 2 hours	3.06 ± 2.30	4.31 ± 2.51	
VAS 24 hours	2.46 ± 2.27	3.79 ± 2.74	
*Primary outcome*			
Change in pain intensity (ABS) at 1 hour, compared to baseline	−3.46 ± 2.32 ***p < 0.0001	−1.78 ± 1.89 ***p < 0.0001	***p < 0.0001
Change in pain intensity (REL) at 1 hour, compared to baseline	−59% ± 35%	−30% ± 31%	***p < 0.0001
*Secondary outcomes*			
Change in pain intensity (ABS) at 2 hours, compared to baseline	−2.87 ± 2.24 ***p < 0.0001	−1.85 ± 1.96 ***p < 0.0001	*p = 0.028
Change in pain intensity (REL) at 2 hours, compared to baseline	−50% ± 36%	−32% ± 37%	*p = 0.026
Change in pain intensity (ABS) at 24 hours, compared to baseline	−3.46 ± 2.65 ***p < 0.0001	−2.38 ± 2.27 ***p < 0.0001	*p* = 0.062
Change in pain intensity (REL) at 24 hours, compared to baseline	−57% ± 37%	−40% ± 40%	*p = 0.037
**Rescue medication use**			
*Secondary outcomes*			
Patients having used rescue medication at 2 hours	3 (6)	2 (4)	*p* = 0.66
Patients not having used rescue medication at 2 hours	44 (94)	50 (96)	
Patients for whom medication data were not available at 2 hours	5 (10)	2 (4)	
Patients having used rescue medication within 24 hours	18 (40)	21 (41)	
Patients not having used rescue medication within 24 hours	27 (60)	30 (59)	*p* = 1
Patients for whom medication data were not available within 24 hours	7 (13)	3 (6)	
**Pain-free and pain relief data**			
*Supplementary results*			
Patients reporting pain freedom at 1 hour	15 (29)	3 (6)	**p = 0.0016
Patients reporting pain freedom at 2 hours	9 (17)	4 (7)	*p* = 0.15
Patients reporting pain freedom at 24 hours	15 (29)	7 (13)	*p* = 0.056
Sustained pain freedom for 24 hours	3 (6)	0 (0)	*p* = 0.11
Patients reporting ≥30% pain relief at 1 hour	41 (79)	21 (39)	***p < 0.0001
Patients reporting ≥30% pain relief at 2 hours	34 (65)	28 (52)	*p* = 0.17
Patients reporting ≥30% pain relief at 24 hours	38 (73)	33 (61)	*p* = 0.22
Sustained ≥30% pain relief for 24 hours	20 (38)	11 (20)	*p* = 0.055
Patients reporting ≥50% pain relief at 1 hour	33 (63)	17 (31)	**p = 0.0017
Patients reporting ≥50% pain relief at 2 hours	28 (54)	22 (41)	*p* = 0.24
Patients reporting ≥50% pain relief at 24 hours	31 (60)	26 (48)	*p* = 0.25
Sustained ≥50% pain relief for 24 hours	15 (29)	8 (15)	*p* = 0.10

Data are expressed as mean ± standard deviation or *n* (%).

ABS: absolute value; REL: relative percentage.

* *p* < 0.05; **p < 0.01; ***p < 0.0001.

**Figure 5.**
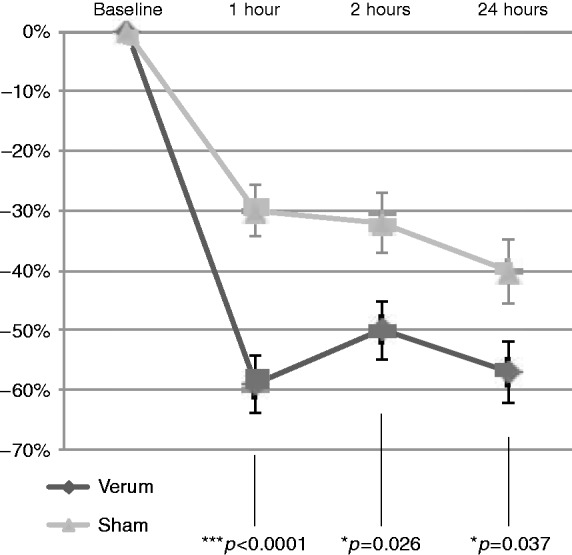
Relative change in mean VAS scores at 1 hour, 2 hours, and 24 hours after treatment, compared to baseline.

The mean pain score reduction at the 2-hour time point compared to baseline was also greater in the verum group compared to the sham group (−50% vs −32%) and reached the level of statistical significance (*p* = 0.026). Mean pain score reduction at the 24-hour time point compared to baseline was likewise greater in the verum group compared to the sham group (−57% vs −40%), reaching statistical significance (*p* = 0.037). The evolution of the relative pain reduction after 1 hour, 2 hours and 24 hours compared to baseline is depicted in Figure 5. With respect to rescue treatment, 18 patients in the verum group and 21 patients in the sham group used rescue medication within 24 hours (difference was not significant).

Regarding our exploratory endpoints, the proportion of pain-free subjects was significantly higher in the verum group (29%) than in the sham group (6%) at the 1-hour time point (*p* = 0.0016), but not at the 2- and 24-hour time points. The proportion of subjects achieving ≥50% pain relief was significantly higher in the verum group than in the sham group at the 1-hour time point (63% vs 31%, *p* = 0.0017) but not at the 2-hour and 24-hour time points. The proportion of subjects achieving ≥30% pain relief was significantly higher in the verum group compared to the sham group at the 1-hour time point (79% vs 39%, *p* < 0.0001), but not at the 2- and 24-hour time points.

When correcting the *p*-values for multiple comparisons using the Holm method, the proportion of subjects reporting pain freedom, of subjects achieving ≥30% pain relief, and of subjects achieving ≥50% pain relief at 1 hour remained significantly higher in the verum group (*p* = 0.027, *p* = 0.0016, and *p* = 0.027, respectively). However, none of the secondary outcomes reached statistical significance when correction for multiple comparisons was applied.

A modified intention-to-treat (mITT) post hoc analysis was additionally performed including only compliant patients (i.e. those who completed the 1-hour treatment phase and provided pain scores at baseline and at the 1-hour time point). A total of 99 patients were included in this mITT analysis (verum: *n* = 47; sham: *n* = 52). In the mITT analysis, the primary outcome measure was significantly more reduced in the verum group (−65%) than in the sham group (−32%; *p* < 0.0001). The secondary and exploratory outcomes in the mITT analysis were similar to those in the ITT analysis. Notably, the difference in the proportion of subjects who were pain-free at the 24-hour time point (32% vs 13%, verum vs sham), and who had ≥30% sustained pain relief (43% vs 21%), were statistically significant (*p* = 0.032 and *p* = 0.030, respectively).

A subgroup analysis was also performed for those patients who did not use any acute medications for their qualifying migraine attack at any point prior to enrollment and administration of e-TNS. Among these 70 patients, the mean VAS score reduction at 1 hour was significantly greater in the verum group than in the sham group (−3.6 ± 1.7 vs −1.8 ± 1.9, *p* < 0.0001).

With respect to migraine subgroups, there was a statistically significant difference in pain reduction between verum and sham for the ‘migraine without aura’ attacks: mean VAS reduction at 1 hour was −3.3 ± 2.4 for the verum group versus −1.7 ± 1.9 for the sham group (*p* = 0.0006). For the ‘migraine with aura’ attacks, pain reduction was numerically greater for verum versus sham, but did not reach significance: mean VAS reduction at 1 hour was −4.3 ± 1.8 for the verum group versus −2.6 ± 1.9 for the sham group (*p* = 0.060).

Regarding safety, there were no serious adverse events (SAEs) and no adverse device effects (ADEs) reported throughout the course of the study. In terms of minor AEs, three patients (two in the verum group and one in the sham group) were unable to tolerate the paresthesia sensation during the nociceptive threshold test phase (before the first 5 minutes of stimulation elapsed), and the treatment was stopped before proceeding to the full stimulation phase. Four patients (three in the verum group and one in the sham group) discontinued treatment before the end of the full stimulation hour: among the patients who received verum stimulation, one withdrew due to nausea that was not present before the beginning of the session (which subsequently resolved without intervention after 20 minutes) and the other two patients discontinued treatment shortly after the initial stimulation test phase as they sensed the paresthesias to be painful. There were no other adverse effects or subjective complaints reported for either group within the 24 hours after the beginning of the treatment.

## Discussion

Previous open-labeled pilot studies have suggested a role for e-TNS in the acute treatment of migraine (16–18); however, evidence-based efficacy data are lacking. The ACME study is the first randomized, sham-controlled trial of e-TNS for the acute treatment of migraine, specifically investigating whether a 1-hour session of e-TNS is both safe and effective in providing headache relief during a migraine attack. We will first review the results and shortcomings of this trial, then discuss the clinical significance of our findings in the context of currently available abortive migraine therapies, and finally comment on the possible mechanisms of action of e-TNS in the acute treatment of migraine.

This study met the primary outcome measure, indicating that e-TNS (using the previously specified stimulation parameters) is more effective than sham stimulation for the acute treatment of migraine. Although there was no difference in rescue medication intake following e-TNS compared to sham (which could argue for a lack of clinical efficacy), the verum device demonstrated significantly superior pain relief compared to sham at the 1-hour, 2-hour, and 24-hour time points (discounting pain scores following any rescue medication intake). The proportion of pain-free patients was also significantly higher for the verum device at the 24-hour time point in the mITT analysis. In a recent observational survey (18), patients using e-TNS reported avoiding the use of acute migraine medications in 42.6% of their migraine attacks. A possible explanation for the lack of difference in rescue medication intake in our current study is that subjects were not given any instructions after the e-TNS session regarding their acute migraine medication use. Consequently, subjects may have taken their usual acute medications to treat residual mild headache and/or for fear of potential headache recurrence. In pharmacologic acute migraine clinical trials (23,24), subjects are often instructed that they may take their usual acute rescue medication if they have persistent or subsequently develop moderate-to-severe headache; in some trials, use of triptan medications is not even permitted (23).

The efficacy results in the current study were more robust than reported in prior pilot studies (16,17) that used shorter e-TNS sessions (20 minutes and 30 minutes). Therefore, it appears that the duration of treatment is an important parameter, with a longer session resulting in better pain relief. In the present study, the mean pain score in the active treatment group was lower at the 1-hour time point than at the 2-hour time point, a finding that was also observed in our previous open-labeled pilot trial (19) with a 1-hour e-TNS session and may suggest that the treatment effect is short-lasting in some patients. Additional investigations with longer e-TNS sessions are thus of interest to determine the most appropriate protocol for acute migraine treatment.

With regards to limitations of our study, there was a small sample size (powered based on calculated assumptions noted earlier) and unbalanced baseline characteristics between the verum and sham groups for migraine type, migraine duration, and prior acute medication use. These differences in baseline characteristics were subsequently accounted for in a post hoc ANCOVA analysis, without modifying the significance of the treatment effect defined by the primary outcome.

Another limitation of the current study was the execution in a clinic setting. This was designed to ensure correct application of the electrode and device (supervised by study staff) and to maximize data compliance, as the primary outcome measure was collected on site. However, the results could differ from the ‘real-world’ context and may not be generalizable to use of e-TNS at home. In addition, application of e-TNS at the clinic may enhance the placebo effect – pain reduction for the sham group (−30% at 1 hour) is higher than published in a previous pharmacologic acute migraine study (21) (−10% at 1 hour), though the difference in delivery system should be noted (device versus oral placebo). Despite this placebo rate, the primary outcome in our study remained highly significant between the two treatment groups, reinforcing the efficacy of e-TNS.

Regarding safety, there were no severe SAEs and one subject in the active treatment group reported nausea during stimulation that was self-limited. In addition, e-TNS was generally well tolerated. When used for migraine prevention outside a migraine attack, approximately 2% of patients reported intolerance to the paresthesia sensation of e-TNS (8). As e-TNS was applied here during a migraine attack, we hypothesized a higher incidence of intolerance to the forehead paresthesia induced by the stimulation due to increased cutaneous allodynia and low nociceptive threshold during a headache (25), which could render the treatment impractical. However, only three patients (two in the verum group and one in the sham group), or 2.8% of total subjects, were unable to pass the nociceptive threshold test (the first 4 minutes of stimulation), and another four patients (three in the verum group and one in the sham group) discontinued stimulation before completion of the 1-hour treatment as mentioned above, yielding a total of seven patients out of 106 (6.6% of total subjects) who could not or did not comply with the treatment session. The safe and well-tolerated nature of e-TNS constitutes an advantage over standard acute migraine medications, and offers an alternative for patients who have contraindications to or experience adverse effects with triptans or NSAIDs. In contrast to pharmacologic acute treatments, e-TNS also does not bear the risk of causing medication overuse headache or chronification of migraine, as frequent use of e-TNS may actually result in a preventive effect (7).

Another potential role for e-TNS is the acute treatment of prolonged and/or medically refractory migraine. It is known that acute medications including triptans, the mainstay of acute migraine treatment, are less effective if taken late into a migraine attack (beyond 1 hour of headache onset) and when pain intensity is moderate–severe compared to mild (26,27). In the current study, e-TNS was effective even when applied late into a migraine attack, and also in cases where patients had failed to respond to standard oral migraine medications (taken more than 3 hours prior). This points to the possible utility of e-TNS in the emergency room setting, where some migraine patients present if their headache fails to improve with standard outpatient acute medications, or as a strategy to obviate the need for such emergency room visits.

Direct comparison of efficacy to standard acute migraine treatments (e.g. triptans and NSAIDs) is limited by differences in trial design. Our objective here was to determine whether e-TNS is effective in providing acute headache relief, while the primary endpoint in the triptan trials is typically defined as the proportion of pain-free subjects at the 2-hour time point, as recommended by the International Headache Society guidelines for controlled trials of migraine drugs (20). Furthermore, in most pharmacologic acute migraine trials, headache intensity is measured on a four-point scale from 0 (no headache) to 3 (severe headache). Here, we used an 11-point VAS score that would provide more sensitivity for our primary outcome measure: change in headache severity at 1 hour. In addition, the setting of treatment administration differs, with e-TNS being applied in the clinic setting in the current study and acute migraine medications being taken at home. Nevertheless, one study reported reduction in mean pain VAS scores at 1 hour of 26.8% for diclofenac 100 mg and 17.1% for sumatriptan 100 mg (21), which is notably less than the reduction of 59% observed with e-TNS in our study. The treatment effect at 1 hour was 7.1% for sumatriptan 100 mg and 16.8% for diclofenac 100 mg, which is also notably less than the 29% treatment effect observed here for e-TNS. At 2 hours, mean pain score reductions were 44% for diclofenac and 56% for sumatriptan, compared to 50% with e-TNS. While these comparisons are encouraging, additional randomized controlled trials using the same protocol design as the triptan abortive trials are needed for a better understanding of the therapeutic positioning of e-TNS in the acute treatment of migraine. Furthermore, it would be interesting to assess the efficacy of e-TNS when used early in a migraine attack, (i.e. at the onset of headache when pain intensity is still mild, as studied in the ‘early intervention’ triptan trials).

The mode of action of e-TNS in migraine is not fully understood; it may have segmental ‘gate control’ mechanisms, as well as supra-segmental actions. Scarce evidence for a segmental mechanism comes from a pilot study in which amplitude of the nociceptive blink reflex (nBR) was transiently reduced after one 20-minute e-TNS session (16). A single session of e-TNS in migraine patients during an attack relieves pain, but has no effect on cerebral metabolism (10). Conversely, after several months of e-TNS used daily for migraine prevention, there is an increase in metabolism assessed with FDG-PET in pre-treatment hypo-metabolic medial prefrontal cortical areas, including the anterior cingulate cortex (9), while trigeminal noxious heat-induced functional magnetic resonance imaging (fMRI) blood oxygen level-dependent (BOLD) hyper-activation of the latter normalizes (10). These metabolic changes are accompanied clinically by a significant decrease in monthly attack frequency in compliant patients. A recent sham-controlled trial furthermore showed that one 20-minute session of e-TNS reduces the amplitude of laser heat-evoked cortical potentials, especially in the anterior cingulate cortex (11). Taken together, the available preliminary evidence suggests that the anterior cingulate gyrus might be a crucial hub on which eTNS could act to alleviate migraine headache.

## Conclusion

The results from this multi-center, randomized, double-blind, sham-controlled trial demonstrate that e-TNS is effective for acute pain relief during migraine attacks in adult subjects, providing a reduction of mean pain intensity at 1 hour of 59% (vs 30% in the sham group, *p* < 0.0001). e-TNS is safe and well tolerated, offering migraine patients a non-invasive, acute treatment option that lacks the systemic side effects associated with conventional migraine medications.

## Clinical implications

- This is the first randomized, double-blind, sham-controlled clinical trial evaluating the safety and efficacy of e-TNS for the acute treatment of migraine attacks with or without aura.
- Use of e-TNS during a migraine attack provided a significant reduction in mean headache pain intensity at 1-hour, 2-hour, and 24-hour time points compared to sham stimulation.
- The acute treatment of migraine with e-TNS was safe and well tolerated.
